# Complete assembly of a dengue virus type 3 genome from a recent genotype III clade by metagenomic sequencing of serum

**DOI:** 10.12688/wellcomeopenres.14438.2

**Published:** 2019-01-10

**Authors:** Mary Dias, Chitra Pattabiraman, Shilpa Siddappa, Malali Gowda, Anita Shet, Derek Smith, Barbara Muehlemann, Krishnapriya Tamma, Tom Solomon, Terry Jones, Sudhir Krishna

**Affiliations:** 1 St. John's Medical College and Hospital, Bangalore, 560034, India; 2 National Institute of Mental Health and Neurosciences, India, Bangalore, 560029, India; 3 Centre for Cellular and Molecular Platforms, Bangalore, 560065, India; 4 Trans-Disciplinary University, Foundation for Revitalization of Local Health Traditions, Bangalore, 560064, India; 5 Johns Hopkins Bloomberg School of Public Health, Baltimore, Maryland, 21205, USA; 6 Center for Pathogen Evolution, Department of Zoology, University of Cambridge, Cambridge, CB2 3EJ, UK; 7 World Health Organization Collaborating Center for Modeling, Evolution, and Control of Emerging Infectious Diseases, Cambridge, CB2 3EJ, UK; 8 Indian Institute of Science, Bangalore, 560012, India; 9 Institute of Infection and Global Health, and National Institute for Health Research, Health Protection Research Unit in Emerging and Zoonotic Infections, University of Liverpool, Liverpool, L69 7BE, UK; 10 National Centre for Biological Sciences, Tata Institute of Fundamental Research, Bangalore, 560065, India

**Keywords:** DENV3, metagnomics, febrile illness

## Abstract

**Background:** Mosquito-borne flaviviruses, such as dengue and Japanese encephalitis virus (JEV), cause life-threatening diseases, particularly in the tropics.

**Methods:** Here we performed unbiased metagenomic sequencing of RNA extracted from the serum of four patients and the plasma of one patient, all hospitalized at a tertiary care centre in South India with severe or prolonged febrile illness, together with the serum from one healthy control, in 2014.

**Results:** We identified and assembled a complete dengue virus type 3 sequence from a case of severe dengue fever. We also identified a small number of JEV sequences in the serum of two adults with febrile illness, including one with severe dengue. Phylogenetic analysis revealed that the dengue sequence belonged to genotype III. It has an estimated divergence time of 13.86 years from the most highly related Indian strains. In total, 11 amino acid substitutions were predicted for this strain in the antigenic envelope protein, when compared to the parent strain used for development of the first commercial dengue vaccine.

**Conclusions:** We demonstrate that both genome assembly and detection of a low number of viral sequences are possible through the unbiased sequencing of clinical material. These methods may help ascertain causal agents for febrile illnesses with no known cause.

## Introduction

Acute undifferentiated febrile illness refers to a sudden onset of high fever without localized organ-specific clinical features ^1^. Although the majority of patients recover over a few days, some can develop severe illnesses, resulting in high morbidity and even death in many parts of the world. Among the many causes of febrile illness, some of the most important across Asia are mosquito-borne viruses such as dengue virus ^1– 6^. In addition, novel agents associated with acute febrile illness continue to be discovered ^7– 9^.

Current molecular diagnostic techniques, such as polymerase chain reaction, are pathogen-specific and therefore pose limitations, as they may fail to detect co-infections and novel agents not commonly associated with the disease syndrome ^10^. The unbiased metagenomic sequencing of clinical material from patients with acute fever can overcome these limitations ^3, 11^.

Mosquito-borne viruses of the family *Flaviviridae*, which include dengue virus and Japanese encephalitis virus (JEV) are known to co-circulate in India and other parts of Asia ^12^. Dengue viruses are a major cause of acute febrile illness in Asia, with recurrent outbreaks having occurred ^13^. JEV, on the other hand, is better known as a cause of acute encephalitis ^14^. Although JEV has been noted as an agent that causes acute fever in Southeast Asia, it is not routinely tested as a cause of fevers in India ^5, 6^. There are four distinct serotypes of dengue viruses (DENV1–DENV4), with their small RNA genomes (approximately 10.8 kbp) making them amenable for characterization by deep sequencing of infected mosquitoes or clinical material from infected individuals ^15^. Sequencing dengue genomes is important for tracking virus evolution, given that they frequently mutate ^15, 16^. Outbreaks of severe dengue disease associated with serotype switches or the introduction of a novel strain into the population have been reported from several different countries, including Sri Lanka, Pakistan and Singapore ^17– 22^. Recent analysis suggests an influenza-virus-like pattern for dengue virus evolution, where strain-specific differences underlie antibody neutralization ^23^. Pre-existing antibodies to circulating dengue strains can therefore contribute to disease severity by inadequate neutralization of the virus or by antibody-mediated enhancement, which facilitates virus infection ^24– 28^. This is supported by *in vitro* studies, which found that changes to the envelope (E) protein of DENV3 were sufficient to alter antibody binding ^26^. Multiple dengue vaccines are currently in various stages of development, and a tetravalent vaccine (CYD-TDV; Dengvaxia®, Sanofi Pasteur) has been approved for use in several countries ^29, 30^. This vaccine has been shown to induce the expression of broadly neutralizing antibodies to multiple strains and all serotypes of dengue viruses ^31^. The results of a phase III trial of this vaccine suggest that both the immune state (with respect to dengue viruses) and circulating viruses may influence vaccine effectiveness ^29^. This underscores the need to characterize both the sequence evolution and antibody response of circulating dengue strains.

Here we used an unbiased sequencing/metagenomic approach, in order to determine both the identity and sequences of viruses associated with febrile illness. In particular, based on previous studies of sequencing data from the serum of febrile individuals, we expected that medium-depth sequencing (about 10–20 million sequence reads per sample) was necessary and sufficient to provide complete sequences of small viral genomes from clinical material ^2, 9^. To test this, we sequenced RNA extracted from the serum of four individuals and the plasma of another presenting with febrile illness at a tertiary care hospital in Bangalore, India and one healthy control from the same hospital, during the dengue season of 2014. We recovered the complete coding sequence of DENV3 clustering into a recent genotype III clade.

## Results

**Figure 1.**
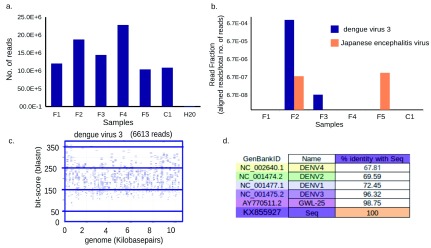
Dengue virus type 3 (DENV3) and Japanese encephalitis virus sequences identified from febrile serum. ( **a**) Number of sequence reads generated per sample. ( **b**) Bar graph showing number of reads that aligned to a particular virus as a fraction of the total number of reads ( *y*-axis, log scale) from that sample ( *x*-axis) using the SNAP alignment. ( **c**) Alignment of sequences mapping only to DENV3 by nucleotide BLAST. Each rectangle shows sequencing reads (blue lines), their alignment to the genome ( *x*-axis) and their blast bit-score ( *y*-axis). Numbers below the title represents number of reads that mapped to the title. ( **d**) Percentage identity of KX855927 with all four dengue viruses and the closest Indian strain.

We sequenced RNA extracted from the serum of four patients hospitalized with severe febrile illness and from one plasma sample from a patient hospitalized with prolonged febrile illness ( Table 1). We included serum from a healthy individual and water as controls. Approximately 10×10 ^6^ sequence reads were recovered from each sample, with the water control yielding a lower number of reads ( Figure 1A).

**Table 1.** Clinical Profile of the sequenced cases. The clinical presentation, key diagnostics tests, provisional diagnosis, treatment followed and results from sequencing (SNAP alignment against viral databases) are shown.

Sample	Age/sex	Presentation	Investigations	Diagnosis	Management	Animal viruses (sequencing+ BLAST)
F1	34F	Fever, vomiting, loose stools, hypotension	• dengue IgM + • Serial platelet count: 57,000-12,000- 37,000-60,000 cells/mm ^3^ • BP 106/72 mmHg	Dengue	Platelet transfusion, antiemetics, IV fluid; patient recovered and was discharged after 5 days	None matched
F2	28F	Fever, severe myalgia for 4 days, hypotension	• Dengue IgM + • Dengue NS1 + • LFT: AST 370 U/l; ALT 170 U/l; GGT 272 U/l • Chest X ray: Bilateral pleural effusion • Serial platelet count: -7000-16000- 43000 cells/mm ^3^ • BP 80/60mmHg	Dengue	Platelet transfusion IV fluids; patient improved and was discharged	Dengue virus 3 (19,120 reads) Japanese encephalitis virus (14 reads)
F3	36F	Fever and severe myalgia for 15 days	Weil–Felix border line positive (OX K 1:80) for Rickettsial fever	Rickettsial fever	Doxycycline (200 mg for 7 days); patient recovered	None matched
F4	10M	Prolonged fever (>20 days)	No known cause	Provisional diagnosis Rickettsial or partially treated enteric/malaria		dengue virus 3 (1 read)
F5	42F	Fever for 13 days, chills and rigors, known diabetic	Weil–Felix suggestive of Rickettsial Fever (OX K 1:320)	Rickettsial fever	Doxycycline (200 mg for 5 days); patient improved	Japanese encephalitis virus sequences (12 reads)

M, Male; F, Female; IgM, dengue immunoglobin M; NS1, dengue non-structural protein 1 test; LFT, liver function test; AST, aspartate aminotransferase; ALT, alanine aminotransferase; GGT, gamma glutamyltransferase.

A BLAST ^32^ similarity search, mapping all sequenced reads to a database of NCBI reference viral sequences ( Table 1), identified 19,120 DENV3 sequence reads and 14 JEV sequence reads in sample F2, and 12 JEV sequence reads in sample F5. A single DENV3 read was detected in sample F3. No animal viruses were confirmed by BLAST in the controls or in other samples ( Table 1 and Figure 1B).

On the basis of World Health Organization guidelines for the classification of dengue cases ^33^, F2 was classified as a case of severe dengue, as the presenting symptoms included respiratory distress (bilateral pleural effusions in chest X-ray) hypotension and elevated liver enzymes ( Table 1).

The serum sample from this individual was positive for both the non-structural protein 1 antigen and dengue IgM, and we were able to obtain a complete DENV3 genome sequence from this sample. Genomes were assembled both by *de novo* (87.05% coverage) and mapping-based (99% coverage) assembly ( Table 2 and Table 3, Supplementary File 1) and found to be identical ( Supplementary File 2). Mapping revealed good coverage across the genome, with an average depth of 231.45 ( Figure 1, Table 2). The genome is missing 76 bp at the 5’-UTR and 28 bp at the 3’-UTR compared to the NCBI RefSeq ( NC_001475.2) DENV3 genome.

**Table 2.** Assembly characteristics for mapping based assembly. The quality, coverage and percentage nucleotide identity of the assembled DENV3 genome using different back bones and sequences for mapping using MIRA assembler are shown.

Criteria	Backbone	av.qual	#-reads	mx.cov.	av.cov	GC%	CnNoCov
All Reads from F2 against all 4 Refseq of dengue viruses	DENV3	41	2009	96	26.27	46.67	145
	DENV1	30	2	3	1.01	46.67	10587
	DENV2	30	1	1	1	45.82	10723
	DENV4	30	1	1	1	47.12	10649
“virus reads” from F2 against all 4 Refseq of dengue viruses	DENV3	42	18180	788	231.53	46.66	104
	DENV1	30	3	4	1.02	46.67	10587
	DENV2	30	1	1	1	45.82	10723
	DENV4	30	1	1	1	47.12	10649
“virus reads” from F2 against DENV3 and an Indian strain	DENV3 (RefSeq)	42	18178	793	231.51	46.66	104
	AY770511.2	43	18696	792	236.58	46.65	104

Table shows the quality, coverage and percentage nucleotide identity of the assembled DENV3 genome using different back bones and sequences for mapping using MIRA assembler. Backbone, reference genome used for assembly; av.qual, average quality of assembly; mx.cov, maximum coverage of assembled genome by reads; av.cov, average coverage of assembled genome by reads; No cov, number of nucleotides of reference not covered in assembly; DENV3, dengue virus type 3.

**Table 3.** Assembly characteristics for *de novo* assembly. The assembly characteristics by *de novo* assembly of sequences from sample F2 after quality assessment was performed using the QUAST tool.

Fraction of genome covered	Largest alignment	Total aligned length	% nucleotide identity with mapping assembly	Reference for quality
87.046	**3127**	9403	**100.00%**	Refseq DENV3

DENV3, dengue virus type 3.

The mapping-based assembly was used for phylogenetic analysis and submitted to GenBank, with accession number KX855927. The degree of nucleotide identity between this strain and the reference DENV3 genome (NC_001475.2) was 96.32%, and with the closest DENV3 strain from India, 98.75%.

**Figure 2.**
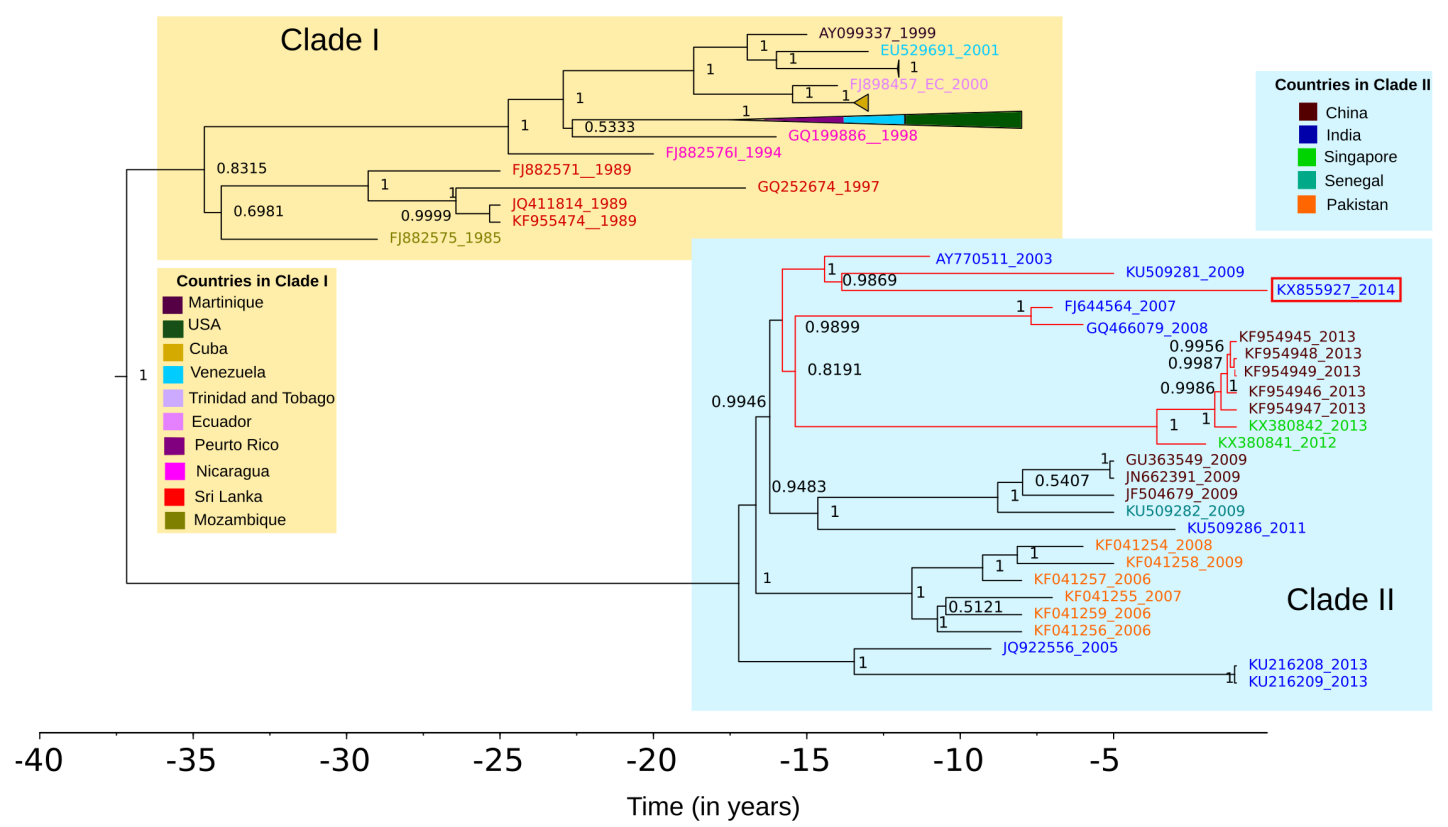
The sequenced strain KX855927 (2014) belongs to a recent Asian clade within genotype III. Figure shows the BEAST maximum clade credibility tree of the top 79 BLAST matches to KX855927 The Indo–China–Singapore strain to which KX855927 (2014) is shown in red. All strains are represented by their GenBank IDs and coloured by country. For ease of visualization, a clade containing viruses from the USA, Venezuela and Puerto Rico in Clade I has been collapsed (pyramids colored by country). The *x*-axis represents time in years.

Phylogenetic analysis was carried out with BEAST2 using the coding sequence of KX855927 and 79 sequences selected as being similar to KX855927, using the BLAST search against dengue genomes in the Virus Pathogen Database and Analysis Resource ^34^ ( Supplementary File 3). The strain clusters with recent DENV3 sequences from India, China and Singapore ( Figure 2). This clade split from other DENV3 and other DENV3 genotype III strains around 15 years ago. The branch length of KX855927 is longer than most others in the tree, with an estimated divergence time of 13.86 years (with the 95% highest posterior densities between 12.94 and 14.83 years) from the closest Indian strain ( Figure 2). A maximum likelihood tree showed the same topology as the consensus tree from BEAST, although many clades had low bootstrap support ( Supplementary File 4).

**Figure 3.**
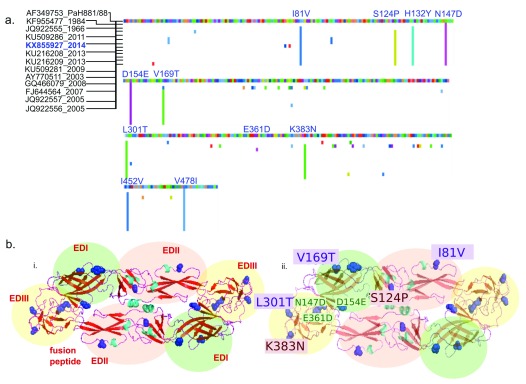
Shared amino acid substitutions in the envelope protein of Indian DENV3 strains differ from PaH881/8. ( **a**) Multiple sequence alignment of region coding for the envelope (E) protein of dengue virus 3 from India were aligned to gi|13310784|gb|AF349753.1| DENV3 strain PaH881/88 polyprotein precursor, translated E genes. Suffix represent the year of sampling. Predicted amino acid changes compared to PaH881/88 are shown in colour. Position of substitutions present in the sequenced KX855927 strain are shown in blue. ( **b**) i) Cartoon structure of E protein KX855927 (2014)- dimer, homology modeled in SWISS-PROT with the domains shaded green (EDI), pink (EDII) and yellow (EDIII), labeled in red. ii) Cartoon structure of E protein KX855927 (2014)- dimer, homology modeled in SWISS-PROT showing the amino acid substitutions in KX855927 (2014) compared to the PaH881/8 in one of the dimers. In both cartoons, predicted substitutions are shown in blue (side-chains colored). Amino acid substitutions labelled in violet (violet box) are positions known to influence mouse monoclonal antibody binding. Positions in red (red box) are among 32 positions in the E protein predicted to be important for antigenicity.

Both synonymous and non-synonymous substitutions were predicted throughout the genome, as compared to the DENV3 reference sequence ( Supplementary File 5). We aligned the E protein of all the complete genomes from Indian strains against the parent strain used to derive the tetravalent dengue vaccine (CYD-TDV; Dengvaxia®, Sanofi Pasteur) ( Figure 3). Multiple amino acid substitutions were predicted throughout the envelope protein and two additional stop codons (at amino acid positions 58 and 168) were observed in the DENV3 KX855927. Most of the amino acid substitutions were shared among all the Indian strains, while a E361D substitution was unique to the DENV3 strain reported here ( Figure 3A). Of the substitutions, 9 out of 11 were mapped onto the surface of the E protein. Of these, six are in key antigenic sites, with three sites known to influence antibody binding ( Figure 3B).

The sequencing reads mapping to JEV from Sample F2 and F5 were assembled into contigs and used to check for potential alignment to other genomes in the NCBI nucleotide sequence database. A BLAST search revealed that the JEV sequences we identified were specific to JEV (100% identity, 100% coverage of read) ( Supplementary File 6). The sequences were found to match non-structural protein 5 of JEV. A specific search against the dengue database for the contigs from the sample containing DENV3 sequences showed no similarity for contig 1 and some similarity to a dengue virus 2 sequence for contig 2 (83% identity, 97% coverage; Supplementary File 6).

The single DENV3 sequencing read found in sample F3 was identical to a sequencing read occurring with high frequency in sample F2. Therefore, we did not carry out any further analysis with this sequence read as we suspect it to be a contamination.

## Discussion

Here we sequenced a complete dengue genome from a clinical case of severe dengue fever, without the need to culture the virus, and in an unbiased manner. We believe that, in the future, the sequence-based -enrichment of viral sequences using conserved sequences, will enable the recovery of complete genomes from routine clinical samples even with by lower-depth sequencing ^35^.

We identified a low number of reads mapping specifically to JEV. JEV is known to cause fevers ^5, 6, 36^. Further systematic analysis using a combination of polymerase chain reaction and IgM testing is required to ascertain how much JEV contributes to the acute fever burden in India. The low number of JEV reads obtained in both samples in which reads mapping to JEV were found suggests there was not much active viral replication occurring. There are previous reports of the detection of JEV sequences many months after infection ^37^. The sequences we found could therefore be remnants of a previous infection or may be the result of an infection from a mosquito bite that was checked by the immune system. The low number of reads in these cases mapped to the same gene (non-structural protein 5) ( Supplementary File 6). This could reflect the higher stability of some parts of the JEV RNA genome.

The results of metagenomic sequencing, however, do need to be interpreted with caution owing to issues related to contamination ^10, 11^. Contamination can occur in every step of the procedure, starting from sample collection, processing, sequencing and, when multiple indexed samples are sequenced together, de-multiplexing (the process in which reads get assigned to a sample). This needs to be taken into consideration, particularly when the number of sequences supporting the presence of a pathogen are low, when there is incomplete genome information, or when the same sequence is present in all the samples, including the controls. We have tried to mitigate this partially by the use of controls—serum from a healthy individual collected at the same time and place and a water sample processed in the same way as the clinical samples. However, we believe that independent methods are required to confirm novel/unexpected findings by this method.

DENV3 has been shown to be re-emerging in India, and has been responsible for severe outbreaks in other geographic regions, including in South America and Cuba ^27, 38, 39^. The full-length DENV3 (KX855927) we describe here clusters into a clade containing DENV3 viruses from India and is related to an Indo–China–Singapore clade. We observed a longer branch length for this particular strain, which could be the result of incomplete sampling of this clade or could indicate that this lineage is showing accelerated rates of molecular evolution ^40^. This can be resolved in future studies by the addition of more sequence information, as more full-length dengue sequences from India become available in the databases.

While both synonymous and non-synonymous changes were observed throughout the DENV3 (KX855927) genome compared to the DENV3 reference sequence (NC_001475.2), the changes in the antigenic E protein are of particular interest. Neutralizing antibodies have been described against the envelope protein that target particular epitopes ^26, 41^. Critical amino acid residues that change antibody binding have also been described by others ^26^. The results from our phylogenetic analyses are consistent with previous work tracing the emergence of new clade of DENV3 genotype III strains in India ^39^. The ability of a dengue vaccine to elicit neutralizing antibodies against locally circulating DENV3 strains therefore needs to be evaluated in this light.

## Methods

### Description of samples

In total, samples from five patients (two diagnosed with dengue fever (serum; F1 and F2), two with Rickettsial fever (serum; F3 and F5) and one with unknown fever (plasma; F4) presenting with febrile illness, and one healthy control (serum; C1) at St. John’s Medical College and Hospital (SJMCH), Bangalore, were assessed in this study. Table 1 provides clinical characterization, treatment and outcomes of patients. The study was done after obtaining approval from the Institutional Ethics Committee of SJMCH, Bangalore, India (IEC Study Ref. No. 5/2016). A waiver of consent was sought and obtained for the analysis as it was done on samples remaining after routine diagnostic testing, which were de-linked from the identity of the patients. We have been granted a waiver of consent by the Institutional Ethics Committee of St. John’s Medical College and Hospital, which does not permit the use of the generated data for human genetic studies.

### Isolation of RNA

RNA was extracted using the Qiagen All-Prep kit, using 300–500 µl of serum/plasma and lysed using 1 ml of lysis buffer. The remaining protocol was performed as recommended by the manufacturer. Eluted RNA was concentrated and used for sequencing reactions.

### Sequencing

Sequencing libraries were prepared using the Ion Proton library preparation protocol. Indexing was performed using the IonXpress RNA Seq Barcode kit (Thermo Fisher Scientific, Inc.). Samples F1–4 and C1 were run on the same chip; sample F5 was run on a separate chip. Libraries were pooled to give equimolar concentrations of 10 pM. This was used in template-preparation steps and RNA sequencing was performed using the Ion PI sequencing kit on the Ion Proton platform using the Ion PI™ ChipV2 and Ion PI™ Sequencing Kit V3 (Thermo Fisher Scientific, Inc.).

### Analysis of sequences

We aligned the sequencing reads to a database of all known viruses using the SNAP alignment tool (snap-1.0beta.16-linux) ^42^. All hits were verified using nucleotide BLAST sequence search and visualized using tools from the Dark Matter project. Reads aligning to the human genome, human mRNA, rRNA large subunit and rRNA small subunit from the SILVA database were removed ^43^. The aligned sequences were used as the input for assembly. *De novo* assembly was performed using the SPAdes (v3.10.1) tool ^44^. Quality assessment of the assembly was performed using the QUAST tool ^45^. MIRA 4.0.2 was used for mapping based assembly, with the GenBank sequence NC_001475.2 for DENV3 as the backbone for assembly and NC_001437 as the backbone for JEV ^46^. Contigs were subjected to nucleotide BLAST using the online BLAST tool. The mapping based assembly of DENV3 obtained using MIRA was manually checked for regions with low confidence using Gap5 (staden-2.0.0b11-2016-linux-x86_64) ^47^. Fewer than 30 nucleotides were found to have low confidence, of which 22 were in the 3’-UTR end region. The files from the MIRA assembly, together with the contributing reads, are provided as Supplementary File 1. This sequence was submitted to GenBank with the accession number KX855927.

### Phylogenetic analysis

Phylogenetic analysis was performed with BLAST search hits to KX855927 in the VipR dengue virus database ^34^. Only the coding sequence was used for the analyses. The alignment was visualized using AliView software (v1.18) ^48^. Nucleotide distances of KX855927 from other dengue viruses, using the reference sequence and the closest BLAST hit from India, were estimated using the MUSCLE alignment tool to create a percentage identity matrix ^49^. The Generalized Time Reversible Model, namely GTR+I+G, GTR+I+G, GTR+G, were found to be the best evolutionary models for codon positions 1, 2, and 3, respectively, using PartitionFinder (v2.1.1) ^50^, where I represents invariant and G represents gamma, a shape parameter for the model. A previously estimated rate of substitution for DENV3 =7.48×10 ^−4^ subs/site/year (4.47×10 ^−4^; 10.72×10 ^−4^) was used to set a strict molecular clock ^51^. The input XML file to BEAST (v2.4.6) ^52^ is provided in Supplementary File 3. Tracer (v1.6) was used to confirm sufficient sampling (effective sample size > 200 for all parameters). TreeAnnotator (v2.4.6) was used to generate the maximum clade credibility tree, where the node heights represent median height. Posterior probabilities for both the split of Clade I and II and the clade containing KX855927 were >95%. The tree was visualized using FigTree (v1.4.3). The Maximum Likelihood tree was generated using thorough search and 1000 bootstraps in RaXML (RAxML -NG v0.4.1 BETA) ( Supplementary File 4) ^53^.

### Analysis of E protein

E protein alignments for the DENV3 complete genomes from India were performed in AliView and amino acid differences were highlighted compared to PaH881/8 (AF349753) the parent strain used in the development of Dengvaxia (CYD-TDV; a tetravalent, live attenuated, chimeric dengue vaccine with a yellow fever 17D backbone). Homology modeling was performed for the E protein of KX855927 using SWISS-MODELSWISS-MODEL and the best model was chosen for showing the substitutions. The protein surfaces, as visualized using PyMOL (version 1.8; PyMOL Molecular Graphics System, Schrödinger, LLC), are shown in light brown; the amino acids found to be different in the KX855927 strain are colored by the CHNOS elements. The datasets supporting the conclusions of this article are included within the article and in Supplementary File 1– Supplementary File 6.

An earlier version of this work can be found on bioRxiv ( https://doi.org/10.1101/204503).

## Data availability

The raw files from sequencing are not provided in their entirety as these are metagenomic datasets that contain identifying host information. Therefore we have used only sequences not aligning to the human genome for our research. This data has been uploaded in fastq format on OSF (see below). As our experiments were designed to identify pathogens, we do expect the accompanying human data to be free from biases involving sampling, storage and handling. However, under the conditions that the samples remain de-identified, and the work is not directly on human genetics, approval for data sharing of the complete data from the RNA sequencing experiment, which includes any human sequences, can be sought with the Institutional Ethics Committee, St. John’s Medical College and Hospital, Bangalore. A request for use of this data for a research proposal must be submitted to the ethics committee via the lead author ( soniamarydias@hotmail.com).

Fastq files have been made available from OSF, http://doi.org/10.17605/OSF.IO/RMQDF ^54^.

Data are available under the terms of the Creative Commons Zero “No rights reserved” data waiver (CC0 1.0 Public domain dedication).

## Supplementary material

**Supplementary File 1: Dengue virus type 3 assembly with contributing reads.** Consensus sequence and reads contributing to the assembly, it contains regions/bases flagged by MIRA including 30 base positions which have low confidence. The file format (.maf) can be converted to compatible file formats for viewing with Gap5, Consed and other genome editors.

**Supplementary File 2: Percentage identity of dengue virus type 3 sequence assembled by different methods.** Table shows the percentage similarity between the *de novo* and mapping assemblies compared to NCBI reference sequences of dengue virus types 1-4 and the closest Indian strain.

**Supplementary File 3: Template for BEAST.** The input file used for phylogenetic analysis using the BEAST program.

**Supplementary File 4: Maximum Likelihood Tree from RaxML.** Figure shows the maximum likelihood trees with bootstrap values, GenBank IDs and year of sequencing are shown on the tips. The data is coloured by country and some of the clades have been collapsed for ease of viewing.

**Supplementary File 5: Single nucleotide polymorphisms in KX855927 with respect to NC_001475.** Description of the single nucleotide polymorphisms in KX855927 with respect to NC_001475, as detected by the MIRA assembly program.

**Supplementary File 6: BLAST results of Japanese encephalitis virus contigs from sample F2 and F5.** Contains the Top 5 nucleotide BLAST lists for the Japanese encephalitis virus contigs assembled from samples F2 and F5, against the nucleotide database, flavivirus database and dengue virus database.
