# Supplementary figures and images for: Complete assembly of a dengue virus type 3 genome from a recent genotype III clade by metagenomic sequencing of serum
Source: Wellcome Open Res. 2019 Jan 10;3:44. Originally published 2018 Apr 23. [Version 2] doi: 10.12688/wellcomeopenres.14438.2 (PMC6085601; doi:10.12688/wellcomeopenres.14438.2)

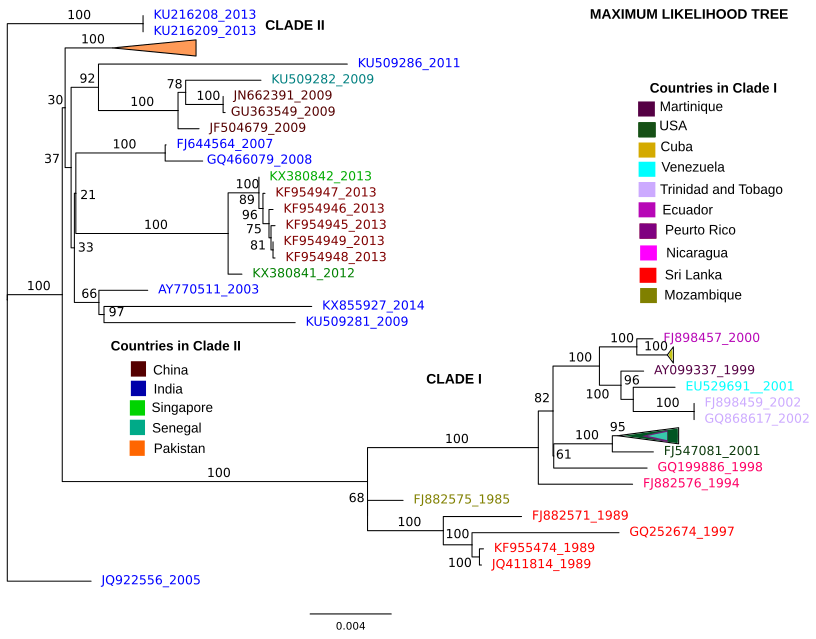

Supplement: Supplementary file 4 [file wellcomeopenres-3-16381-s0003.tgz › c5bbc782-9d08-4cca-8e17-0f19757bd52a.png]
