# Supplementary material for: Complete assembly of a dengue virus type 3 genome from a recent genotype III clade by metagenomic sequencing of serum
Source: Wellcome Open Res. 2019 Jan 10;3:44. Originally published 2018 Apr 23. [Version 2] doi: 10.12688/wellcomeopenres.14438.2 (PMC6085601; doi:10.12688/wellcomeopenres.14438.2)
Supplement: Supplementary file 2 [file wellcomeopenres-3-16381-s0001.tgz › a11c0931-ce08-4ba0-b6af-42fb6f3ef7d8.doc]

**Supplementary File 2 - Percentage identity of DENV3 sequence assembled by different methods**

All sequences were clipped to align with the scaffold sequence from de novo assembly using SPAdes and joined using ScaffoldBuilder (<http://edwards.sdsu.edu/scaffold_builder/>). The clipped sequences were used as input for the MUSCLE alignment program which also generates a percentage identity matrix. The results from the analysis are tabulated above as percentage nucleic acid identity. Values to the right of the diagonal which are identical to the values of the left have been removed for ease of viewing. Map -all- reads represents the assembly when all reads were directly used as input for MIRA, de novo represents the scaffold resulting from SPAdes and Scaffold Builder and map-virus-reads reads represents the mapping based MIRA assembly from reads that aligned to viruses using the SNAP alignment tool. DENV1-4 represent the 4 dengue serotypes, GWL-25 represents the closest Indian strain to the assembled strain.
