# Supplementary material for: Complete assembly of a dengue virus type 3 genome from a recent genotype III clade by metagenomic sequencing of serum
Source: Wellcome Open Res. 2019 Jan 10;3:44. Originally published 2018 Apr 23. [Version 2] doi: 10.12688/wellcomeopenres.14438.2 (PMC6085601; doi:10.12688/wellcomeopenres.14438.2)
Supplement: Peer review history

# Peer review: Referee response for version 1

**DOI:** 10.21956/wellcomeopenres.15716.r33558

**Published:** 2018-08-08

**Authors:** John A Lednicky

**Affiliations:** 1 Department of Environmental and Global Health, College of Public Health and Health Professions, Emerging Pathogens Institute, University of Florida, Gainesville, FL, USA

The report is straight-forward and presents a successful application of metagenomics sequencing to help diagnose febrile illnesses with no known causes. The assembled DENV3 sequence will be very useful for epidemiology studies and to track the evolution of DENV3.

 Specific items for the authors to consider:

1. OPTIONAL: On page 3, the authors mention Dengvaxia.  It will actually strengthen the authors’ projected use of metagenomics sequencing if they mentioned the problems in the Philippines and elsewhere with this vaccine. Will it help to know before vaccines are used which virus strains are in circulation? And will that sequence knowledge be useful in the future for evaluating the possible side-effects of vaccines before the vaccines are administered?
2. Figure 3: As viewed on my computer and a printed copy of the manuscript, neither blue nor violet colors were discernable for FIG. 3b.
3. OPTIONAL: The authors might clarify: On page 7, DISCUSSION, par. 1: It is not clear if the authors agree with the reference cited (35), or whether they have experienced this first hand.
4. Page 8: Isolation of RNA:  Why was the All-Prep kit chosen for serum/plasma instead of a QIAGEN viral RNA kit?  The former is typically used for whole blood.  How was the eluted RNA concentrated?  Why?
5. OPTIONAL:  The authors make no mention of the costs/time commitments/ personnel/training/facilities to accomplish the type of work they performed. Though the approach is very good (and clearly the wave of the future), it is at present very costly and not practical for most medical laboratories, even in developed countries.  How can this be remedied? 
6. OPTIONAL: The authors might comment further on this: It will be curious to many readers why JEV sequences were also detected.  Is this a finding of major importance?  Has this been observed in other studies (persistence of JEV sequences)? Could the severity of illness in patient F2 have been affected by a “smoldering” JEV infection?
7. More explanation would be useful regarding how the sequencing libraries were made. For example, flavivirus RNA is not polyadenylated and is capped.  Do the authors have a specific approach to capture these types of RNA?

*OPTIONAL: Some possible clarifications/comments from the authors:

 (1) Next generation sequencing definitely has a role diagnostics, phylogeographic studies, etc.  On page 3, the authors state “We hypothesized that …”.  Some readers will interpret that to mean the authors claim they conceived this concept, and this is the first time the approach has been used.  The authors might reword that statement. 

 (2) The authors did not comment on the specificity of the DENV IgM test.

 (3) Many NGS-derived virus sequences deposited in data banks have errors.  Thus, whereas the NGS-generated data is useful for the diagnosis, indels etc. are not always verified in the submitted sequences, the general understanding among users being that errors may be present.  Also, often, the 5’ and 3’ UTRs are not included. The authors might discuss these issues.  In particular, there are artefacts associated with Ion Proton sequencing associated with repeats of the same nucleotide (example: TTTTT).

I have read this submission. I believe that I have an appropriate level of expertise to confirm that it is of an acceptable scientific standard.

# Response

**Published:** 2019-01-03

**Authors:** Chitra Pattabiraman

**Affiliations:** National Institute of Mental Health and Neurosciences, India, India

*We thank the reviewer for the comments. They are addressed point-wise below.*

*1. New Generation sequencing approaches are attractive options to identify viral quasispecies in patients. In the present article, the authors have presented a common consensus final sequence of DENV-3 and compared it with reference sequence and the closely related GWL-25 genome. Did the authors had a chance  to look at the raw read clusters, and identify possible variations that could represent quasi species by looking at sites/ sequence regions showing consistent variability?* *A comparison table showing the non-synonymous sites could have been included.*

We have not performed a systematic analysis for viral variants within our sample, however, given that we got greater that 100X coverage of many regions of the genome, we believe this analysis is possible. A comparison table showing the changes between the DENV3 strain reported here and the RefSeq DENV3 strain are included in Supplementary File 5. 

2. *Considering that DENV virus protein translation starts as a polyprotein formation from a single ORF, identifying two internal stop codons at positions 58 & 168 seems to be curious. Have the authors done a manual verification of the corresponding raw read alignments? What could be the implications of this finding*?

We have realigned the sequence and manually verified this. Both changes are well supported. Our experiment cannot distinguish between defective viral particles and viable ones. Also, we are potentially putting together sequences from different viruses to make the consensus. These two factors limit our ability to interpret these findings. 

3. *The authors have identified two missing regions-one each in the 5' and 3' UTR. Are they internal gaps or terminal truncation in the sequence? Could this be a failure to sequence these regions by the technique or is it possible that the DENV-3 strain itself is lacking these regions? Since the UTRs contain major replication regulatory elements, discussing this observation seems to be important.*

Given the importance of the UTRs in viral replication, we strongly suspect that our approach has limitations in capturing the ends of the genome. 

4. *In the result section, the unique substitution is mentioned as D361E, where as in the fig.3A it is shown as E361D. The reader gets confused on the order of the sequences that are being compared.*

The textchas been corrected in line with the figure. 

Most of the amino acid substitutions were shared among all the Indian strains, while a D361E substitution was unique to the DENV3 strain reported here (Figure 3A).

to

Most of the amino acid substitutions were shared among all the Indian strains, while a E361Dsubstitution was unique to the DENV3 strain reported here (Figure 3A).

5. *In Fig.3b(ii), the authors have highlighted the amino acid residues that are important in antibody binding and antigenicity. But how many of these changes are functionally important, based on the chemical nature of the amino acid substitutions observed? Also, the authors should indicate the wild type as well as the mutant amino acids along with the position number in the figure.*

The figure and accompanying legend have been modified. 

6. *Was there any specific reason in using plasma sample from one patient and serum from others? It would be good to indicate, if available, the duration of fever in F1, and treatment and outcome in F4, for reasons of consistency.*

In this study, we used samples remaining after routine testing and plasma sample was available for that patient.

# Peer review: Referee response for version 1

**DOI:** 10.21956/wellcomeopenres.15716.r33079

**Published:** 2018-05-17

**Authors:** Easwaran Sreekumar, Shijulal Nelson-Sathi

**Affiliations:** 1 Computational Biology & Bioinformatics, Rajiv Gandhi Centre for Biotechnology-BIC Campus, Kinfra, Kazhakoottam, Thiruvananthapuram, Kerala, India; 2 Molecular Virology Laboratory, Rajiv Gandhi Centre for Biotechnology (RGCB), Thiruvananthapuram, Kerala, India

The manuscript by Dias *et al* describes the use of metagenomic sequencing of RNA from  serum/plasma  samples of febrile patients using an Ion Proton system to identify viral sequences. Using this approach they could identify presence and complete sequence of DENV-3 in one of the samples tested. They could also detect presence of Japanese Encephalitis virus sequences in the sample from the same patient and also in another sample. The work is interesting and has shown the possibility of using this approach in clinical samples for detection of pathogen sequence signatures.

 This reviewer had the following queries:

 1.New Generation sequencing approaches are attractive options to identify viral quasispecies in patients. In the present article, the authors have presented a common consensus final sequence of DENV-3 and compared it with reference sequence and the closely related GWL-25 genome. Did the authors had a chance  to look at the raw read clusters, and identify possible variations that could represent quasi species by looking at sites/ sequence regions showing consistent variability?

 A comparison table showing the non-synonymous sites could have been included.

 2. Considering that DENV virus protein translation starts as a polyprotein formation from a single ORF, identifying two internal stop codons at positions 58 & 168 seems to be curious. Have the authors done a manual verification of the corresponding raw read alignments? What could be the implications of this finding?

 3.The authors have identified two missing regions-one each in the 5' and 3' UTR. Are they internal gaps or terminal truncation in the sequence? Could this be a failure to sequence these regions by the technique or is it possible that the DENV-3 strain itself is lacking these regions? Since the UTRs contain major replication regulatory elements, discussing this observation seems to be important.

 4.In the result section, the unique substitution is mentioned as D361E, where as in the fig.3A it is shown as E361D. The reader gets confused on the order of the sequences that are being compared.

 5.In Fig.3b(ii), the authors have highlighted the amino acid residues that are important in antibody binding and antigenicity. But how many of these changes are functionally important, based on the chemical nature of the amino acid substitutions observed? Also, the authors should indicate the wild type as well as the mutant amino acids along with the position number in the figure.

 6.Was there any specific reason in using plasma sample from one patient and serum from others?

 It would be good to indicate, if available, the duration of fever in F1,  and treatment and outcome in F4, for reasons of consistency.

We have read this submission. We believe that we have an appropriate level of expertise to confirm that it is of an acceptable scientific standard.

# Response

**Published:** 2019-01-03

**Authors:** Chitra Pattabiraman

**Affiliations:** National Institute of Mental Health and Neurosciences, India, India

We thank the reviewers for the comments. They are addressed point wise below

*1. New Generation sequencing approaches are attractive options to identify viral quasispecies in patients. In the present article, the authors have presented a common consensus final sequence of DENV-3 and compared it with reference sequence and the closely related GWL-25 genome. Did the authors had a chance  to look at the raw read clusters, and identify possible variations that could represent quasi species by looking at sites/ sequence regions showing consistent variability?* *A comparison table showing the non-synonymous sites could have been included.*

We have not performed a systematic analysis for viral variants within our sample, however, given that we got greater that 100X coverage of many regions of the genome, we believe this analysis is possible. A comparison table showing the changes between the DENV3 strain reported here and the RefSeq DENV3 strain are included in Supplementary File 5. 

2. *Considering that DENV virus protein translation starts as a polyprotein formation from a single ORF, identifying two internal stop codons at positions 58 & 168 seems to be curious. Have the authors done a manual verification of the corresponding raw read alignments? What could be the implications of this finding*?

We have realigned the sequence and manually verified this. Both changes are well supported. Our experiment cannot distinguish between defective viral particles and viable ones. Also, we are potentially putting together sequences from different viruses to make the consensus. These two factors limit our ability to interpret these findings. 

3. *The authors have identified two missing regions-one each in the 5' and 3' UTR. Are they internal gaps or terminal truncation in the sequence? Could this be a failure to sequence these regions by the technique or is it possible that the DENV-3 strain itself is lacking these regions? Since the UTRs contain major replication regulatory elements, discussing this observation seems to be important.*

Given the importance of the UTRs in viral replication, we strongly suspect that our approach has limitations in capturing the ends of the genome. 

4. *In the result section, the unique substitution is mentioned as D361E, where as in the fig.3A it is shown as E361D. The reader gets confused on the order of the sequences that are being compared.*

The texthas been corrected in line with the figure. 

Most of the amino acid substitutions were shared among all the Indian strains, while a D361E substitution was unique to the DENV3 strain reported here (Figure 3A).

to

Most of the amino acid substitutions were shared among all the Indian strains, while a E361Dsubstitution was unique to the DENV3 strain reported here (Figure 3A).

5. *In Fig.3b(ii), the authors have highlighted the amino acid residues that are important in antibody binding and antigenicity. But how many of these changes are functionally important, based on the chemical nature of the amino acid substitutions observed? Also, the authors should indicate the wild type as well as the mutant amino acids along with the position number in the figure.*

The figure and accompanying legend have been modified. 

6. *Was there any specific reason in using plasma sample from one patient and serum from others?*

*It would be good to indicate, if available, the duration of fever in F1, and treatment and outcome in F4, for reasons of consistency.*

In this study, we used samples remaining after routine testing and plasma sample was available for that patient.
